# Kinase-targeted therapy in subsets of colorectal cancer

**DOI:** 10.18632/oncoscience.580

**Published:** 2023-06-27

**Authors:** Patricia M. Gomez Barila, Jan Paul Medema

**Keywords:** colorectal cancer, kinase inhibitors, signaling pathways, drug resistance

Colorectal cancer (CRC) is one of the most commonly diagnosed cancers and the second leading cause of cancer-related deaths worldwide [1]. Early diagnosis and adequate treatment are crucial for improving patient prognosis, although this remains difficult due to the high molecular and clinical heterogeneity of the disease [2]. However, recent efforts have been made to stratify CRC patients and uncover novel targeted therapies for patient groups with a poor response to available chemotherapy [3].

One of the CRC classification systems involves the identification of key pathways that are dysregulated due to genetic mutations or differential cellular wiring. Important known pathways in CRC include the Wnt, MAPK, PI3K and p53 pathways [4]. Kinases are the proteins responsible for carrying out the signal transduction within the pathways, leading to particular cellular phenotypes, such as increased proliferation and migration [5]. More specifically, higher activity and dysregulation of certain kinases has been widely shown in cancer, with the modulation of kinase activity through available chemical inhibitors leading to successful treatment options for a number of patients. Accordingly, uncovering essential kinases for tumor growth and invasion is crucial in the development of more effective targeted therapy in metastatic or later stage CRC.

Recent research has led to the identification of a number of kinases that are known to play an important role in the development and progression of CRC. Several predictive kinases for targeted therapy have been uncovered, including BRAF V600E mutation, ALK and NTRK fusions [6]. Another important pathway involves the mitogen-activated protein kinases (ERK MAPK), which is located downstream of several growth factors including epidermal growth factor (EGF). Upstream activation of the pathway by RAS-RAF, resulting in increased activity of the ERK/MAPK pathway, is known to play an important part in the development of CRC [7]. Cetuximab, an EGF inhibitor, was the first targeted agent approved by the FDA and has shown to be effective specifically in patients with wildtype RAS and BRAF CRC [3]. Alternative treatment for patients with metastatic BRAF-mutated CRC is still undergoing clinical trials with a focus in combination treatment with kinase inhibitors, since this group of patients does not respond to EGFR inhibition and poorly to chemotherapy alone.

Patient response to kinase inhibitors may also be dependent on the cross-talk between certain pathways, since preventing the activity of a signaling cascade may also lead to unfavorable effects. For example, patients who show anti-EGFR resistance under cetuximab treatment also show rapid compensative activation of the IGF-1, PI3K, JAK, VEGF or HER2 pathways. In order to circumvent the resistance to cetuximab, preclinical studies have tested combination therapy with BRAF/EGFR/PI3K inhibitors, which led to clinical trials showing some response but only in a small group of patients [8]. Additional efforts to target anti-EGFR resistance involve the blockage of HER2, which is shown to be amplified in 5% of metastatic CRC. Several clinical trials have shown promising response to HER2 blockade and have used HER2 amplification as a biomarker for anti-EGFR resistance [9]. Although some research is being focused on the use of kinase inhibitors in CRC, the cross-talk between the pathways remains an issue in resistance to available treatment. Another important factor to consider when investigating kinase inhibition is the biological heterogeneity of CRC. It has been shown that there is a strong variation in the dependency on specific kinases for distinct subtypes of CRC. For instance, a subgroup of CRC with poor-response to chemotherapy does not appear to respond to cetuximab treatment either even when the tumors are wildtype for RAS and RAF [10, 11]. In the future, multiple tumor biological features should be considered when stratifying patients and identifying optimal kinase inhibitor treatment options since the biological heterogeneity of CRC tumors translates to differences in therapy response.

At the pre-clinical level, screening methods including drug response, RNAi, CRISPR-Cas9 and bioinformatical gene expression analyses have been useful in unveiling key kinases and novel potential therapy targets for CRC subtypes. For example, we have used the consensus molecular subtypes (CMS), recapitulated *in vitro*, to uncover sensitivities for each of the subtypes using a CRISPR-Cas9 drop-out screen focused on the kinome. We identified PAK2 to be a key kinase in invasion and proliferation for CMS4, which is the poor-prognosis and mesenchymal subtype of CRC. Additionally, we unveiled a number of kinases that require further validation for the other subtypes, with CMS2 (the epithelial and Wnt active subtype) showing sensitivity to the largest number of kinases [12]. In a different study, we used a bioinformatical analysis of gene expression from both tumor and *in vitro* models that had been previously CMS-classified. We successfully identified AKT3 to be solely expressed in CMS4 and uncovered its key role in proliferation, highlighting the fact that the PI3K/AKT/mTOR pathway is crucial for a particular subgroup of CRC tumors [13]. Kranenburg et al. also focused on the CMS4 subtype and identified PDGFRa,b and cKIT as potential selective kinases. Subsequently, they showed in a proof of concept study that Imatinib (cKIT/PDGF receptor inhibitor) reverted the mesenchymal features of CMS4 cancers, suggesting a switch from CMS4 to CMS2 [14]. A different screening approach performed recently featured a combination of CRISPR technology and drug interference to identify targets that may limit the efficacy of BRAF/MEK inhibitors in CRC. They successfully identified a few specific genes (MCL1, BCL2L1 and YAP1) that, when targeted, could sensitize BRAF mutant cell lines to BRAF/MEK inhibitors [15]. We have summarized some of our findings regarding the response of various subsets of CRC to kinase inhibitors in Table 1.

**Table 1:** Response of various subgroups of CRC to kinase inhibitors at clinical and preclinical levels

Kinase – Inhibitor	CRC subgroup	Response
ERK/MAPK – Cetuximab	1. Wildtype Ras and BRAF 2. CMS4 3. CMS2 4. CRIS-C	1. Yes 2. No 3. Yes 4. Yes
cKIT/PDGF receptor – Imatinib	CMS4	Yes
HER2 – Trastuzumab	HER2 amplified mesenchymal	Yes
Group I PAK – FRAX597	CMS4	Yes, preclinical
AKT – MK2206	CMS4	Yes, preclinical
BRAF/MEK – Dabrafenib + Trametinib	BRAF mutant	Yes, preclinical. When MCL1, YAP1 and BCL2L1 are co-targeted

In conclusion, CRC remains one of the deadliest cancer types when diagnosed at later stages due to the biological heterogeneity of the disease resulting in differences in therapeutic efficacy. Efforts to stratify patients based on mutations or gene expression signatures have shown promise in selecting the proper therapy. In agreement, a number of kinase inhibitors have been successfully implemented in the treatment of a specific subgroup of CRC patients. Consequently, further insight into the kinase dependency and the subsequent use of kinase inhibitors in a more subset specific manner will result in improved treatment for CRC patients that have a poor response to available chemotherapy.

## References

[1] Baidoun F, et al. Curr Drug Targets. 2021; 22:998–1009. 10.2174/1389450121999201117115717. 33208072

[2] Markowitz SD, et al. N Engl J Med. 2009; 361:2449–60. 10.1056/NEJMra0804588. 20018966 PMC2843693

[3] Buikhuisen JY, et al. Oncogenesis. 2020; 9:66. 10.1038/s41389-020-00250-6. 32647253 PMC7347540

[4] Fearon ER. Annu Rev Pathol. 2011; 6:479–507. 10.1146/annurev-pathol-011110-130235. 21090969

[5] Hardie DG. Symp Soc Exp Biol. 1990; 44:241–55. 1966636

[6] Dienstmann R, et al. Nat Rev Cancer. 2017; 17:268. 10.1038/nrc.2017.24. 28332502

[7] Fang JY, et al. Lancet Oncol. 2005; 6:322–27. 10.1016/S1470-2045(05)70168-6. 15863380

[8] Punt CJ, et al. Nat Rev Clin Oncol. 2017; 14:235–46. 10.1038/nrclinonc.2016.171. 27922044

[9] Suwaidan AA, et al. Cancer Treat Rev. 2022; 105:102363. 10.1016/j.ctrv.2022.102363. 35228040

[10] Ten Hoorn S, et al. Br J Cancer. 2021; 125:1080–88. 10.1038/s41416-021-01477-9. 34253874 PMC8505637

[11] De Sousa E Melo F, et al. Nat Med. 2013; 19:614–18. 10.1038/nm.3174. 23584090

[12] Buikhuisen JY, et al. J Exp Clin Cancer Res. 2023; 42:56. 10.1186/s13046-023-02600-9. 36869386 PMC9983221

[13] Buikhuisen JY, et al. Cancers (Basel). 2021; 13:801. 10.3390/cancers13040801. 33673003 PMC7918753

[14] Peters NA, et al. Front Oncol. 2022; 12:969855. 10.3389/fonc.2022.969855. 36147916 PMC9486194

[15] Tiedt R, et al. Cell Rep. 2023; 42:112297. 10.1016/j.celrep.2023.112297. 36961816

